# The potential protective role of corticosteroid therapy in patients with asthma and COPD against COVID-19

**DOI:** 10.1186/s12948-021-00159-4

**Published:** 2021-11-01

**Authors:** Fabiana Furci, Marco Caminati, Gianenrico Senna, Sebastiano Gangemi

**Affiliations:** 1 School and Operative Unit of Allergy and Clinical Immunology, Policlinico “G. Martino”, Department of Clinical and Experimental Medicine, University of Messina, Messina, Italy; 2 Department of Medicine, University of Verona and Verona University Hospital, Verona, Italy; 3 Asthma Centre and Allergy Unit, University of Verona and Verona University Hospital, Verona, Italy

**Keywords:** COVID-19, Hospitalization, Asthma, COPD, Corticosteroids, Pulmonary diseases

## Abstract

**Background:**

The observation of patients hospitalized for coronavirus disease (COVID-19) led us to note a lower prevalence of patients affected by chronic respiratory disease, in particular asthmatic patients, compared to the general population. Therefore, the aim of this paper is to evaluate the possible protective role of corticosteroid therapy in patients with chronic lung disease, regarding the risk of contracting severe COVID-19.

**Main body:**

SARS-CoV-2 uses angiotensin-converting enzyme 2 (ACE2) receptors to enter the cells. Considering the high number of these receptors in patients affected by asthma and chronic obstructive pulmonary disease (COPD), the evidence that these patients do not have a high risk of hospitalization for COVID-19 needs further study to understand what the possible protective “factors” are in these patients. In particular, the finding in some studies of reduced coronavirus replication in cell lines treated with steroids, molecules commonly used for treating chronic lung diseases, needs further attention.

**Short conclusion:**

The hypothesis that corticosteroids, commonly used in treating airways diseases, might modify the severity of SARS-CoV-2 disease has become a key point and a possible predictive factor of a positive outcome of COVID-19 in patients treated everyday with these molecules.

## Background

Since the beginning of the COVID pandemic, clinical attention has been paid to analysing the risks of unfavourable outcomes in patients affected by cardiometabolic disease, paying less attention to chronic respiratory disease, although the presence of respiratory diseases might be considered a predictive marker of worse COVID-19 disease severity. To understand the possible relationship between lung diseases and COVID-19, it could be helpful to consider some data present in literature in this regard [1].

In accordance with Li et al.’s study, Caminati et al. reported that hospitalization rates due to COVID-19 in patients with asthma are extremely low, and consistent with asthma prevalence in the general population [2, 3]. Furthermore, taking into consideration the hypothesis that in the case of COVID-19, inhaled corticosteroid (ICS) treatment could modulate response to the SARS-CoV-2 infection, according to the prescribed inhaled treatment, Caminati et al. in the same paper analysed hospital admissions for COVID-19 in asthmatic patients in 2 major hospitals in Verona (Veneto region) and Brescia (Lombardy region), reporting that 5 out of 6 patients with asthma from Verona suffered from moderate asthma; the sixth patient, together with 15 out of 20 patients from Brescia, were all affected by mild asthma, and all reported an optimal adherence rate [3]. Therefore, we aimed to study, analysing literature, if the real reason for the lower incidence of COVID-19 among hospitalized patients, in the presence of respiratory diseases, is in any way due to the role of ICS.

Some studies have reported a reduction of coronavirus replication and cytokine production in cell lines treated with steroids and β-agonists, but other studies reported different data [4–7].

The continuation of ICS therapy by asthmatic patients is important not only for asthma moralanagement, reducing exacerbations and asthma mortality, but, as reported in literature, is also associated with decreased expression of ACE2, the receptor of SARS-CoV-2, in induced sputum. Instead, in subjects without asthma, ICS use does not seem to influence ACE2 expression in lung tissue [8].

The study of the association between pre-existing respiratory disease and its treatment and severe COVID-19 has become the objective of this paper, in order to understand whether steroid therapy may have a modulatory role in the immunological response to COVID-19, and if patients with asthma and COPD could possibly present greater susceptibility to SARS-CoV-2 infection. These data seem surprising, given that respiratory viral infection is the main cause of exacerbations of asthma [9].

## ACE2 expression and ICS therapy: a possible relationship

Maes et al. studied ACE2 mRNA expression in lung tissue in patients affected by asthma and in patients affected by COPD [8]. This expression was significantly increased in (current or former) smokers with COPD, was not altered in subjects with asthma or asthma–COPD overlap (ACO) and pulmonary gene expression of ACE2 did not differ in ICS-treated and non-ICS-treated obstructive airway disease patients. The divergent effects of ICS use on ACE2 expression between both studies might be due to differences in respiratory compartment (induced sputum vs. lung tissue) or patient population (non-smoking patients with asthma vs. smokers with COPD) [8].

## Is there an increased risk of hospital admission with COVID-19 in patients with asthma and COPD?

Aveyard et al. reported that there was no evidence that people with asthma, COPD, and also with bronchiectasis were more likely to be admitted to an intensive care unit (ICU) than people without these conditions [10].

In contrast with this, Aveyard et al. highlighted that patients with respiratory disease using ICS were associated with a modest risk of severe COVID-19, for hospitalization, ICU admission, and for death. Therefore, the authors reported a modestly increased risk of severe COVID-19 in patients with pre-existing respiratory disease, in the representative community cohort they analysed. In particular, people with asthma had an 18% increased risk of hospital admission with COVID-19, not reporting instead an increased risk of COVID-19 death in asthmatic patients [10].

Furthermore, considering the evidence on the association between ICS use and COVID-19, a systematic review reported that, in hospitalized patients affected by severe COVID-19, use of ICS was associated with recovery, and shorter duration of hospital stay, but there were no reported data regarding a possible association with need for ICU care or death [11]. A Spanish cohort highlighted that patients using ICS had a lower risk of hospitalization for COVID-19 than people who did not use ICS [12]. The OpenSAFELY cohort reported that in asthmatic patients there was a lower hospitalization risk for patients using a low or medium dose of ICS, compared to asthmatic patients in therapy with short-acting β-agonist only [13].

These data confirm the role of an anti-inflammatory treatment (principally ICS) in inducing a decline in asthma morbidity, and reducing the utilisation of emergency services, hospitalisations and deaths.

## Corticosteroids: their suppressive role in type 2 inflammation and the restoration of antiviral immunity

Asthma exacerbations, usually induced by a virus, are characterized by augmented airway inflammation, and, consequently, increased respiratory symptoms [14]. Considering that in asthmatic patients there is an inherent impairment of interferon responses to virus infection, use of both inhaled and systemic corticosteroids, which are immunosuppressive agents, can further inhibit production of the critical antiviral mediators type I and III interferons (IFN). In particular, sputum eosinophilia correlates negatively with impaired IFN induction in cultured asthmatic cells and Th2 inflammation (IL-4, IL-13) inhibiting epithelial production of type I interferon [15, 16]. This has been shown in a range of in vitro and in vivo human and animal studies for several asthma-relevant viruses including rhinovirus, influenza, and respiratory syncytial virus (RSV) [17, 18].

These effects lead to increased virus replication and augmented virus-driven pathologies such as mucus hypersecretion and secondary bacterial infections [17, 18]. For example, a study reported that in type I IFN receptor-deficient mice (Ifnar−/−) there is augmented pulmonary eosinophilia and type 2 inflammation in response to influenza infection [19].

In line with this concept, studies reported that in patients with other coronavirus infections (e.g., SARS-CoV-1, MERS-CoV), corticosteroids induce increased viremia and delayed viral clearance [20].

In particular, as reported by Kumar et al. considering that corticosteroids suppress type 2 inflammation, their use in COVID-19 asthmatic patients may lead to the beneficial effect of secondary restoration of impaired antiviral immunity [21]. A previous study reported that inhaled budesonide did not induce an impairment of CD8+ T cell infiltration into the bronchial epithelium following experimental rhinovirus infection in asthmatics, contrary to the clear suppressive effects of corticosteroids on T cells observed in the absence of pre-existing Th2 inflammation [17, 22].

Xu et al. in a recent report of histopathological autopsy from a non-asthmatic patient with COVID-19 treated with systemic corticosteroids, reported suppressed peripheral blood CD8+ T cell numbers [23].

To date, SARS-CoV-2 infection appears to be associated with low levels of blood eosinophils [24].

Although previous studies have reported increased susceptibility to infections in patients treated with ICS, perhaps for a reduction in neutrophil recruitment, it is important to highlight that the effect of ICS may vary depending on the type of respiratory infection, patient factors, such as the severity of the respiratory disease, and the physicochemical properties of ICS [25].

## Anti-inflammatory and immunosuppressive action of corticosteroids

It is important to highlight that whereas the action mechanisms of various ICS is similar, or the binding with the intracellular glucocorticoid receptor, responsible both for genomic and nongenomic effects, they are quite different in terms of receptor affinity, bioavailability, and lipophilicity and drug persistence in the airway is quite different in relation to the pharmacokinetics and characteristics of ICS. With regard to the physicochemical characteristics of fluticasone and budesonide, while fluticasone has a longer duration in airway lumen and mucus, due to poor solubility and permeability across the airway mucosa, budesonide, instead, has greater solubility [26].

Fluticasone, precisely because of its prolonged presence in the airways, leads to a longer glucocorticoid receptor occupancy, to an anti-inflammatory and immunosuppressive action. This is confirmed by a meta-analysis of seventeen trials conducted in 15,336 asthmatic subjects that revealed an increased risk of upper respiratory tract infection with fluticasone but not budesonide and by a meta-analysis of 25 randomized controlled trials involving 49,982 COPD subjects that reported an increased risk of pneumonia with fluticasone but not budesonide [7, 27]. Therefore, the difference between fluticasone and budesonide may be related to the longer retention of fluticasone in the airways, with a potential inhibition of type-1 innate immunity.

The calculation of the equivalent doses of each ICS are available in several equivalence tables, for example, the international GINA guidelines are calculated using the binding affinity, combined with the percent of lung delivery obtained with various formulations (metered-dose inhalers, MDI, vs dry powder inhalers, DPI) available for the different compounds. For instance, 400 mcg of beclometasone in a hydrofluoroalkane (HFA)-propelled pressurised metered-dose inhaler (pMDI) is equivalent to 800 mcg of DPI budesonide and to 500 mcg pMDI/DPI fluticasone. Furthermore, these two compounds have a different lipophilicity (log K): fluticasone (logK¼4.5) and budesonide (logK¼3.7), which may explain the wider distribution of fluticasone [26].

Given that systemic corticosteroids are also used to treat acute exacerbations of asthma, it is possible that this is one mechanism through which people with asthma who are hospitalized with COVID-19 do not have worse outcomes.

## Older age in asthmatic patients: a risk for COVID-19?

As reported by Sunjaya AP older age and the frequent presence of comorbidities in asthmatic patients are strongly associated with a more severe form of SARS-CoV-2 infection, but asthma is not a major risk factor for COVID-19 [28]. Therefore, age can be considered an important predictor for vulnerability to COVID-19 and its prognosis [29, 30].

In accordance with this result, a Spanish study highlighted that asthmatic patients who contract COVID-19 were older and with comorbidities compared to those who were COVID-19 negative. Those older and with comorbidities were also reported to have a higher rate of hospitalization, by 0.23% [12].

## Severe asthma: a possible risk factor for COVID-19?

Williamson et al. analysing 17 million patients with COVID-19, reported an association between severe asthma and increased risk of death for hospitalized patients after adjustment for sex and race, but not for all comorbidities. The hazard ratio was higher for asthma patients with recent oral corticosteroid (OCS) use [31].

Schultze et al. analysed the role of the dose of ICS, highlighting that patients in therapy with high-dose ICS reported an augmented risk of death from COVID-19, by 55%, which was interpreted as being due to confounding by disease severity [13].

Choi et al. reported that asthma severity has a relevant role in inducing poor clinical outcomes of COVID-19: indeed, patients with step 5 asthma had a prolonged in-hospital stay duration than those with step 1 asthma [32]. This data was confirmed by a large COVID-19 hospitalized Italian population that reported the presence of worse COVID-19 outcome (death/need for ventilation vs. discharge at home without requiring invasive procedures) in severe asthmatic patients [33].

## Conclusions

Our analysis suggests that such diseases confer increased risk, but that the extent of this increase in risk, particularly for people with asthma, might be less than originally thought. To what extent up- or downregulation of ACE2 expression in sputum, airways, or lungs has clinical consequences on infectivity or outcomes of COVID-19 needs to be elucidated. In particular, the under-representation of patients with asthma/COPD in COVID-19 may be related to the possible inhibitory effect of ICS on the replication of SARS-CoV-2.

In asthmatic patients, with a proven efficacy of corticosteroids in reducing asthma symptoms and risk of relapse, particularly in those with type 2 inflammation, and restoring antiviral immunity, the use of corticosteroids in the case of SARS-Co-2 infection might be further evaluated according to patient’s condition. Therefore, there is the need to evaluate the risk–benefit assessment of corticosteroid use, both with and without COVID-19 infection, in patients with asthma and COPD, in which ICS or OCS are commonly prescribed.

Therefore, the treatment of asthmatic patients infected with SARS-CoV-2 needs to be personalized, although further studies should be based on the immunopathology of COVID-19-related asthma exacerbation including the extent to which augmented type 2 inflammation drives the pathology.

Maintenance of ICS/OCS in asthma patients is also important to maintain good symptom control, to minimize the risk of an exacerbation and the associated need for hospitalization, which could potentially increase the patient’s risk of SARS-CoV-2 infection. It also important to remember that a poorly controlled respiratory disease can lead to a more complicated disease course for those with SARS-CoV-2 infection.

The invitation to patients affected by respiratory diseases to maintain their regular therapy, for good disease control, preventing potential exacerbation, is the main goal during the COVID-19 pandemic but also in general for a correct model of precision medicine to modulate the immunological response against viruses.

## Data Availability

Not applicable.

## Abbreviations

COVID-19: Coronavirus disease
ACE2: Angiotensin-converting enzyme 2
COPD: Chronic obstructive pulmonary disease
ICS: Inhaled corticosteroid
ACO: Asthma–COPD overlap
ICU: Intensive care unit
IFN: Interferon
RSV: Respiratory syncytial virus
HFA: Hydrofluoroalkane
pMDI: Pressurised metered-dose inhaler
OCS: Oral corticosteroids
